# Internalization of the Thin-Ideal and Eating Pathology in Kuwaiti Adult Women

**DOI:** 10.3389/fpsyg.2020.559711

**Published:** 2020-11-10

**Authors:** Lulwa Zainal, Serena D. Stevens, Jennifer Ann Harriger, Sylvia Herbozo

**Affiliations:** ^1^Social Science Division, Pepperdine University, Malibu, CA, United States; ^2^The Bariatric Center, Cleveland Clinic Akron General, Akron, OH, United States; ^3^Department of Psychiatry and Surgery, University of Illinois at Chicago, Chicago, IL, United States

**Keywords:** thin-ideal internalization, eating pathology, Kuwait, body image, Arab

## Abstract

Rapid socioeconomic growth, Western acculturation, and thin-ideal internalization are theorized to be linked to dieting, weight concerns, and disordered eating in Arab countries. The purpose of this study was to examine eating pathology and the internalization of Western messages regarding the importance of thinness in a community sample of Kuwaiti women. Participants (*N* = 83) aged 18–35 years with BMIs between 17.58 and 24.88 (*M* = 21.30; *SD* = 1.83) completed measures of thin-ideal internalization and eating pathology. Results demonstrated that approximately 28% of the sample was at risk for eating pathology. Thin-ideal internalization was also found to significantly predict eating pathology. Study findings extend prior research on eating pathology in Arabic nations. This study is the first to examine the internalization of the thin-ideal in a community sample of Kuwait adult women and lends further support to the importance of continued research in this population.

## Introduction

Although eating disorders were once considered a Western phenomenon, more recent research has reported dieting, weight concerns, and disordered eating in Arab countries (e.g., Musaiger et al., 2013; Alkazemi et al., 2018). Similar to Western cultures, the rates of disordered eating and body dissatisfaction are generally higher in females compared to males in Arab countries (Musaiger et al., 2013). Researchers cite rapid socioeconomic growth, Western acculturation, and thin-ideal internalization (i.e., the extent to which individuals adopt or “buy into” societal values regarding thinness) as potential reasons for the development of disordered eating in this population (Musaiger and Al-Mannai, 2014; Thomas et al., 2018). However, the majority of published studies examining disordered eating in Arab countries have focused on adolescents (Musaiger et al., 2013) or college students (Thomas et al., 2010, 2018; Alkazemi et al., 2018), which limits the generalizability of the findings; therefore, there is a need for more research on risk factors of eating pathology among female adults in Arab countries.

Musaiger et al. (2013) examined adolescent females from seven Arab countries and reported that the overall rates of disordered eating behaviors in their sample were similar to those reported in Western countries. Additionally, they found that females from Kuwait reported higher levels of disordered eating than those from the other six countries studied (Algeria, Jordan, Libya, Palestine, Syria, and Sharjah). In fact, 42.8% of the Kuwaiti female adolescents met criteria for disordered eating (Musaiger et al., 2013). Another study of Kuwait college women found that 46.4% of the women were at risk for eating pathology (Alkazemi et al., 2018). Openness to Western lifestyle and the influence of Western media were identified as factors that may contribute to body dissatisfaction and the internalization of the thin-ideal in countries such as Kuwait (Musaiger et al., 2013; Alkazemi et al., 2018). Research demonstrates that messages regarding the thin-ideal are prevalent in Western media (i.e., Schaefer et al., 2017), and it is likely that individuals in Kuwait are being exposed to these messages (Alkazemi et al., 2018). However, to our knowledge, no studies have assessed thin-ideal internalization specifically in Kuwaiti females. There has been one study that examined internalization of the thin-ideal in an Arab population. Eapen et al. (2006) studied eating pathology in a sample of adolescent females in the United Arab Emirates and reported that drive for thinness was associated with higher eating pathology. While this study provides initial evidence regarding risk for eating pathology in an adolescent Arab population, it is important to extend the findings to adult females as well.

Additionally, internalization of the thin-ideal is an established risk factor for the development of eating pathology among females in the United States (Thompson and Stice, 2001; Schaefer et al., 2018). The Tripartite model theorizes that one mechanism with which sociocultural pressures (media, parents, and peers) exert their effect on body dissatisfaction and eating pathology (i.e., restriction and bulimic behaviors) is *via* internalization of the thin-ideal. Therefore, it is critical to continue to examine the relationship between thin-ideal internalization and eating pathology in other countries, such as Kuwait, where females may be at an elevated risk compared to females in other Arabic countries (Musaiger et al., 2013).

The purpose of the current study was to extend existing research findings to examine the internalization of the thin-ideal and eating pathology in a community sample of adult females from Kuwait. We hypothesized that females with higher rates of internalization of the thin-ideal would report greater eating pathology. Additionally, given the high rates of eating pathology in adolescent and college female samples from Kuwait (Musaiger et al., 2013; Alkazemi et al., 2018), we also sought to examine the risk of eating pathology in the current sample.

## Materials and Methods

### Participants

Participants included 83 females, ages 18–35 (*M* = 26.30, *SD* = 4.23). Participant BMI ranged from 17.58 to 24.88, with a mean of 21.30 (*SD* = 1.83). All participants were fluent in English and provided informed consent prior to completion of the questionnaires. *Post hoc* power analysis using G*Power 3.1 (Faul et al., 2009), assuming alpha of 0.05 and observed effect size (*f*^2^ = 0.314) indicated a power of 99.3 for this study.

### Procedures

The study was approved by the Internal Review Board at Pepperdine University. Participants were recruited by the first author of this study at community centers and other public centers, such as banks and universities, in Kuwait. The first author approached potential participants and briefly explained the purpose of the study. Interested participants signed a consent form and completed the questionnaires *via* pencil and paper. Data was entered and analyzed in SPSS.

### Materials

#### Demographics

Participants provided demographic information, including their age and height and weight, which were used to calculate BMI.

#### Eating Pathology

Eating pathology was measured *via* the Eating attitudes Test (EAT-26; Garner et al., 1982). The EAT-26 has been widely used to examine eating pathology in both clinical and non-clinical samples (e.g., Gearhardt et al., 2009; Alkazemi et al., 2018). While it has been translated into many languages, the English version has been used and validated in similar Arabic samples (e.g., Thomas et al., 2010). The scale consists of 26 items, which are scored on a six-point Likert scale with responses ranging from 3 (always) to 0 (sometimes, rarely, or never) with possible scores ranging from 0 to 78. Scores greater than 20 are considered to indicate increased risk for eating pathology. The EAT-26 has consistently demonstrated good reliability and validity, and the alpha for the current study was 0.88.

#### Thin-Ideal Internalization

Thin-ideal internalization was measured using the “Internalization: Thin/low body fat” (four items) subscale of the Sociocultural Attitudes Toward Appearance Scale-4R (SATAQ-4R; Schaefer et al., 2017). The scale includes four items. Items are rated on a five-point Likert scale with responses ranging from 1 (definitely disagree) to 5 (definitely agree). Higher scores indicate higher levels of internalization. This scale has demonstrated good reliability and validity (Schaefer et al., 2017), and the alpha for the current study was 0.79.

### Data Analysis

Descriptive statistics were calculated for study variables. A linear regression was conducted, controlling for age and BMI, to assess the hypothesis that thin-ideal internalization would predict disordered eating habits. Thin-ideal internalization was the independent variable and eating pathology was the dependent variable. Age and BMI were entered as covariates.

## Results

Scores on the thin-ideal internalization scale ranged from 4 to 20, with a mean of 12.00 (*SD* = 4.42), and scores on the EAT-26 ranged from 0 to 43, with a mean of 14.06 (*SD* = 11.19). Of the participants, 23 (27.7%) scored above the cutoff score of 20 on the EAT-26, indicating the significant presence of eating pathology. After controlling for age and BMI, the regression model was significant, indicating that 12.1% of the variance in eating pathology can be accounted for by thin-ideal internalization, *F*(3,82) = 6.435 *p* < 0.001, ∆*R*^2^ = 0.121. Specifically, thin-ideal internalization significantly predicted eating pathology, such that for every one SD increase in thin-ideal internalization, eating pathology increased by 0.378 SDs (*β* = 0.378, *t* = 3.45, *p* < 0.001). See Table 1 for model summary.

**Table 1:** Simple linear regression with thin-ideal internalization predicting disordered eating behaviors.

Variable	*Β*	Std. Error	95% CI (*B*)	β	*t*	*p*
(Constant)	3.403	16.451	[−29.343, 36.149]	-	0.207	0.837
Age	−0.504	0.281	[−1.063, 0.054]	−0.191	1.797	0.076
BMI	0.584	0.658	[−0.726, 1.895]	0.096	0.888	0.377
Thin-ideal internalization	0.957	0.277	[0.405, 1.509]	0.378	3.453	0.001

## Discussion

The purpose of the current study was to examine the relationship between the internalization of the thin-ideal and eating pathology in a community sample of women from Kuwait. Interestingly, the percentage of women in our sample that were at risk for eating pathology (27.7%) was lower in our current sample than a sample of Kuwaiti college females (46.4%; Alkazemi et al., 2018) and a sample of Kuwaiti adolescent females (42.8%; Musaiger et al., 2013). In contrast, this prevalence is consistent with research findings in the United States that demonstrate elevated risk for disordered eating in adolescent and college students (Smink et al., 2012).

The hypothesis that women who reported higher rates of thin-ideal internalization would demonstrate increased eating pathology was largely supported, as thin-ideal internalization predicted eating pathology in this sample. This is consistent with research examining populations of young adult women in the United States (Thompson and Stice, 2001) as well as research with adolescent females from the United Arab Emirates (Eapen et al., 2006). Although the results of this study are not necessarily surprising, they do demonstrate the pervasiveness of Western ideals regarding thinness and the risk of eating pathology in Kuwaiti females whom internalize the thin-ideal and support research that demonstrates that Western acculturation is associated with higher eating disorder symptoms in Emirati women (Thomas et al., 2018).

This study has limitations that are important to note. First, this was a pilot study with a small sample size and a limited age range. Future research would benefit from recruiting a larger sample and including participants older than 35 years of age. The cross-sectional design prevents drawing causal conclusions regarding the thin-ideal internalization and eating pathology. Additionally, although the influence of Western ideals *via* the media is considered to be one of the factors affecting body image in Arab nations (Musaiger and Al-Mannai, 2014; Thomas et al., 2018), this study did not specifically measure media exposure. Future research should examine both the content and the outcomes related to media exposure in Arab females, particularly those from Kuwait, to determine it unique effects on body image and eating pathology. Additionally, socio-economic growth and internalization of Western ideals have been cited as potential factors that may be related to disordered eating in Arab populations (Musaiger and Al-Mannai, 2014; Thomas et al., 2018), therefore further examination of these factors in individuals from Kuwait is needed.

To our knowledge, this is the first study to examine the relationship between thin-ideal internalization and disordered eating in a community sample of Kuwaiti women. Study findings extend prior research on eating pathology with female adolescents by focusing on adult women and examining the role of thin-ideal internalization, a strong risk factor for eating disorders (Schaefer et al., 2018). Consistent with prior research in Kuwait females (Musaiger et al., 2013; Alkazemi et al., 2018), eating pathology is prevalent among Kuwait women. Thin-ideal internalization also appears to be contribute to eating pathology in this population as found with women in the United States. This is the first study to demonstrate that internalization of the thin-ideal is related to increased risk of eating pathology in Kuwait females. Study findings provide knowledge that further equips clinicians and researchers to better serve and treat body dissatisfaction and eating disorders in non-Western populations. Intervention strategies shown to decrease thin internalization (Stice et al., 2000) among females in Western populations may be beneficial to utilize with females in Kuwait. Additional research examining thin-ideal internalization and other sociocultural factors contributing to eating pathology in community samples of females from Kuwait as well as other Arab nations is warranted.

## Data Availability Statement

The raw data supporting the conclusions of this article will be made available by the authors, without undue reservation.

## Ethics Statement

The studies involving human participants were reviewed and approved by Seaver College, Pepperdine University IRB. The patients/participants provided their written informed consent to participate in this study.

## Author Contributions

LZ developed the research idea, conducted a literature review, collected the data, and conducted preliminary analyses of the data. SS conducted more advanced analyses and wrote the results section of the manuscript. JH supervised the research project and wrote the introduction, materials and methods, and discussion sections of the manuscript. SH served as a consultant on the project and edited the manuscript. All authors contributed to the article and approved the submitted version.

### Conflict of Interest

The authors declare that the research was conducted in the absence of any commercial or financial relationships that could be construed as a potential conflict of interest.
